# Free-Flap Reconstruction in Early-Stage Squamous Cell Carcinoma of the Oral Cavity—A Prospective Monocentric Trial to Evaluate Oncological Outcome and Quality of Life

**DOI:** 10.3390/jcm12144833

**Published:** 2023-07-22

**Authors:** Julius Moratin, Sven Zittel, Dominik Horn, Rouven Behnisch, Oliver Ristow, Michael Engel, Jürgen Hoffmann, Kolja Freier, Christian Freudlsperger

**Affiliations:** 1 Department of Oral and Cranio-Maxillofacial Surgery, University of Heidelberg, Im Neuenheimer Feld 400, 69120 Heidelberg, Germany; sven.zittel@med.uni-heidelberg.de (S.Z.); oliver.ristow@med.uni-heidelberg.de (O.R.); michael.engel@med.uni-heidelberg.de (M.E.); juergen.hoffmann@med.uni-heidelberg.de (J.H.); christian.freudlsperger@med.uni-heidelberg.de (C.F.); 2 Department of Oral and Cranio-Maxillofacial Surgery, Saarland University Medical Center, Kirrberger Straße, 66424 Homburg, Germany; dominik.horn@uks.eu (D.H.); kolja.freier@uks.eu (K.F.); 3 Institute of Medical Biometry, University of Heidelberg, Im Neuenheimer Feld 130.3, 69120 Heidelberg, Germany; behnisch@imbi.uni-heidelberg.de

**Keywords:** free flap, microvascular reconstruction, survival, early oral squamous cell carcinoma, quality of life

## Abstract

Surgery is generally accepted as standard treatment in oral cancer, but the reconstructive procedures remain a matter of debate. The aim of this study was to evaluate oncological outcome and quality of life following surgical resection and free-flap reconstruction in patients with early oral squamous cell carcinoma. The presented trial was performed as a prospective, single-center observation study. Inclusion criteria were primary surgery in early-stage oral squamous cell carcinoma with free-flap reconstruction. Endpoints were overall and progression-free survival and quality of life up to 24 months after surgery. Twenty-six patients were included. Overall survival was 100% and progression-free survival was 92.3% in a maximum follow-up time of 21 months. Global quality of life showed no significant alteration after surgery. Patients reported a significant reduction in pain (*p* = 0.048) and a decreasing impairment of speech one year after surgery (*p* = 0.021). Free-flap reconstruction is a safe procedure that results in excellent oncological outcome and quality of life. Functional outcome is of high relevance in early-stage tumors of the head and neck and may mostly be affected by reconstructive procedures. Therefore, a prospective evaluation to explore success and the effects of surgical therapy is highly warranted.

## 1. Introduction

Squamous cell carcinoma of the head and neck (HNSCC) is the sixth most common malignancy worldwide with approximately 600,000 new cases every year, and a major part arises in the mucosa of the oral cavity [1,2]. Despite promising developments in diagnosis and stage-adapted multimodal treatments, overall survival rates have remained stable for decades. Especially in advanced tumor stages, overall and progression-free survival is limited [3,4]. While the most important prognostic factors are the extent of the primary disease and the development of regional and distant metastases, the standard therapy with curative intention is surgical tumor resection where achievable in combination with elective neck dissection (END) followed by adjuvant radiation or radio-chemotherapy in patients with pathological risk factors [5,6,7]. Free-flap reconstruction following tumor resection in advanced tumors of the head and neck is generally accepted as standard of care. Nevertheless, the reconstructive approach in early-stage tumors is a matter of debate. According to the reconstructive ladder, primary closure, local flaps, split skin transplants, regional flaps and microvascular flaps are potential technical options for defect closure. In head and neck oncology, the recovery of speech, mastication and swallowing is the major goal in rehabilitation. While data on oncological outcome depending on the operative procedures are limited, many surgeons state that free-flap reconstruction in this cohort may be regarded as overtreatment. Counterarguments include potential flap donor site morbidity, longer surgery times, subsequently prolonged general anesthesia and longer hospitalization if free-flap reconstruction is performed [8]. Considering the generally accepted macroscopic safety margin of 10 mm in all dimensions, relevant defect volumes are caused by tumor ablation, even in small tumors. Neither primary closure nor split skin grafts can restore three dimensional defects sufficiently, and the use of local flaps is limited due to the functional integrity of the oral cavity within a narrow space.

To date, there is a very limited selection of studies on patients with early-stage OSCC and the functional and oncological outcomes following different techniques of reconstruction with contradictory results [9,10,11,12,13]. In order to critically evaluate the oncological results of microvascular reconstruction following tumor ablation in early-stage tumors, we recently conducted a retrospective analysis which proved the concept to be time efficient and safe regarding the oncological outcome of patients. To further enhance the quality of our data and in order to formulate a general treatment recommendation for early-stage OSCC, we established a monocentric cohort study to evaluate the hypothesis in a prospective setting.

Therefore, the aim of this study was to evaluate quality of life and oncological out-come in patients suffering from early-stage squamous cell carcinoma of the oral cavity who underwent surgical tumor resection, elective neck dissection and free-flap reconstruction.

## 2. Materials and Methods

### 2.1. Study Protocol

This prospective study was started in the department of oral and cranio-maxillofacial surgery, University Hospital of Heidelberg, in November 2017. The end of data collection for this interim analysis was October 2020.

Inclusion criteria:

- Histologically confirmed diagnosis of a primary squamous cell carcinoma (SCC) of the oral cavity (stage I or II);
- Clinically negative neck-node status;
- No history of other malignancies;
- Minimum age of 18 years;
- Patient’s approval;
- Ability to understand the character and the consequences of the trial.

Exclusion criteria:

- Advanced clinical tumor stage (Stage III/IV);
- Advanced pathological tumor stage (Stage III/IV);
- Minor patients (age < 18 years);
- Patients with legal assistance;
- Positive history of malignant diseases.

**Figure 1:**
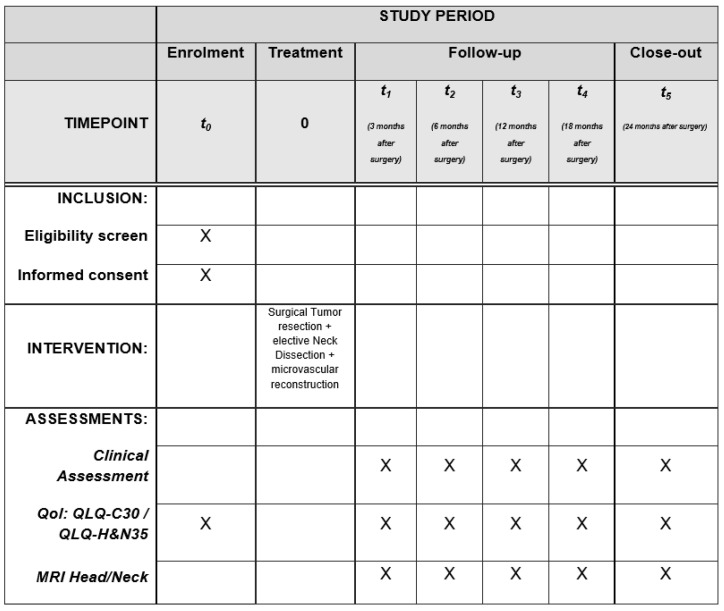
Study timetable with interventions and study related assessment follow-up (“X” marks the appropriate time points in the study protocol).

The study has been conducted in full accordance with ethical principles, including the World Medical Association Declaration of Helsinki (current version), and written informed consent was provided by all patients. Furthermore, the study was approved by the local ethics advisory board (Ethic Vote: S-414/2017) and registered in the German Clinical Trial Register (DRKS00013041). Figure 1 gives detailed information on the study timetable.

Pretreatment diagnostics included panoramic imaging, computed tomography (CT) imaging of the head, neck, and chest and panendoscopy (bronchoscopy and esophagoscopy) to detect possible synchronous malignancies in line with the German National Guideline for oral cancer.

All patients received primary surgical treatment with tumor resection, elective neck dissection and free-flap reconstruction. Patients were re-assessed postoperatively and either stayed in the study or were excluded in dependence of the pathological reports. Reasons for exclusion were advanced tumor stage (T3/T4), incomplete tumor resection (R+), the existence of cervical metastases (N+) or any other reason for adjuvant therapy including close margin resection and histopathological risk factors like perineural or lymphovascular infiltration.

Afterwards, the patients were enrolled in a constant postoperative follow-up in the department’s outpatient clinic. Postoperative imaging using MRI scans were performed to detect local and regional disease recurrence in a predefined fixed interval (3, 6, 12, 18, 24 months after surgery). Relevant data were documented using electronic patient records (SAP, Walldorf, Germany), including clinical and pathological parameters, perioperative data, clinical outcome and postoperative quality of life.

Primary endpoint was progression-free survival after 24 months. Secondary endpoints were overall survival and postoperative quality of life after two years.

Assessment of pre- and postoperative quality of life (QoL) was conducted using well established questionnaires of the European Organization for Research and Treatment of Cancer (EORTC QLQ-C30 Version 3.0 and EORTC QLQ-H&N35 Version 1.0; EORTC Quality of Life Group, Brussels, Belgium). Qol was assessed pre-operatively and 3, 6, 12, 18 and 24 months post-operatively. Scoring was performed as suggested in the publisher’s manual [14,15].

### 2.2. Statistics

Statistical analyses were carried out using Microsoft Excel 2013 (Microsoft, Redmond, WA, USA), SPSS Statistics^®^ 22 (IBM, Armonk, NY, USA) and R (Version 3.6.1). Descriptive data analysis was used to describe epidemiological and perioperative data. Kaplan–Meier survival curves were used to estimate overall and progression-free survival. Overall survival was defined as interval between primary surgery and time of death or last follow-up (censored data). Progression-free survival was defined as interval between primary surgery and time of local, regional, or distant disease progression, death, or last follow-up (censored data). Evaluation of the questionnaires was performed as described previously [16]. Pairwise differences between the scores of each time point were assessed via nonparametric Wilcoxon signed-rank test and the principle of Minimal Clinical Important Difference (MCID) was used to determine relevance [17,18]. Changes of more than 10 points were considered to be clinically relevant [19]. Missing values were imputed via “Person Mean Imputation”.

## 3. Results

### 3.1. Patient Cohort

By the time of the first interim analysis, an overall number of 33 patients had been included in the study. Six patients were excluded after surgery because of advanced pathological tumor stage. One patient decided against a surgical therapy and was excluded consecutively.

The remaining cohort of 26 patients consisted of 16 (61.5%) male and 10 (38.5%) female patients. The ages ranged from 36 to 87 years with a mean age of 66.0 ± 10.8 years. Table 1 gives a summary of demographic, clinical and pathological data of the investigated cohort.

### 3.2. Reconstructive Procedures

**Figure 2:**
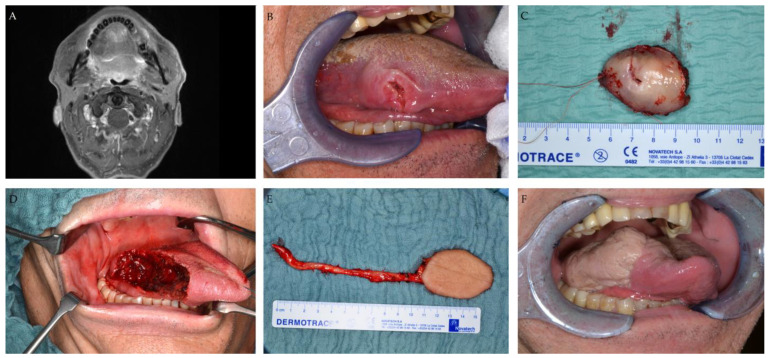
Patient with squamous cell carcinoma of the lateral tongue on the right side. (**A**) MRI scan (T1 sequence) depicting the tumor on the right side of the oral tongue. (**B**) Intraoperative image depicting the tumor. (**C**) Tumor after surgical excision. (**D**) Intraoral defect situation after tumor resection. (**E**) Harvested radial forearm flap. (**F**) Postoperative intraoral situation after tumor resection and reconstruction with radial forearm flap.

All 26 patients received free-flap reconstruction with a radial forearm flap. Microvascular tissue transfer was successful in all patients (Success Rate 100%). Primary closure of donor site was achieved in 4 patients, while donor site defects were closed secondarily (via split skin grafts) or closed by secondary healing in 22 patients after a mean period of 78 ± 39 days. Figure 2 exemplifies the therapeutic course including surgical procedures in a patient with squamous cell carcinoma of the tongue.

### 3.3. Perioperative Data and Time of Hospitalization

Mean surgery time was 306 min. In all cases, a simultaneous two-team-approach was chosen to make procedures efficient. Mean hospitalization time was 13 days.

Table 2 provides information about perioperative morbidity and the duration of hospitalization.

### 3.4. Oncological Outcome

**Figure 3:**
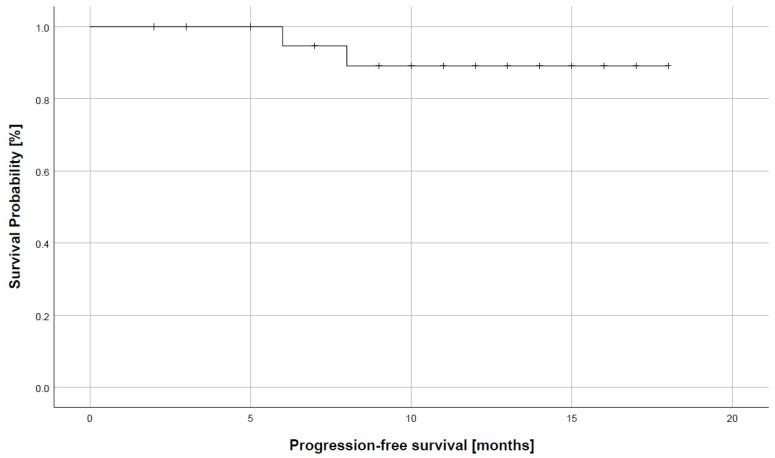
Estimated progression-free survival of the investigated cohort.

By the time of data analysis, all patients were alive (overall survival = 100%). Median follow-up time was 12.5 months. Two patients had developed a local disease recurrence (progression-free survival = 92.3%). Mean time until recurrence was 7.6 months. Both patients were successfully treated via surgical tumor resection and microvascular reconstruction. Figure 3 displays the survival curve regarding progression-free survival for the investigated cohort.

### 3.5. Pre- and Postoperative Quality of Life

The questionnaires were filled in by the patients themselves. All 26 patients completed the baseline questionnaire, 22 patients (84.6%) completed the questionnaires 3 months after surgery, 18 patients (69.2%) completed the questionnaires after 6 months, 13 patients (50%) completed the questionnaires after 12 months, and 3 patients (11.5%) completed the questionnaires after 18 months. Because of the small number of only three patients completing the questionnaires at t4 (18 months after surgery), this analysis mainly compares the scores from baseline to t3. In patients with disease recurrence, the questionnaires were not evaluated after diagnosis of recurrence.

**Figure 4:**
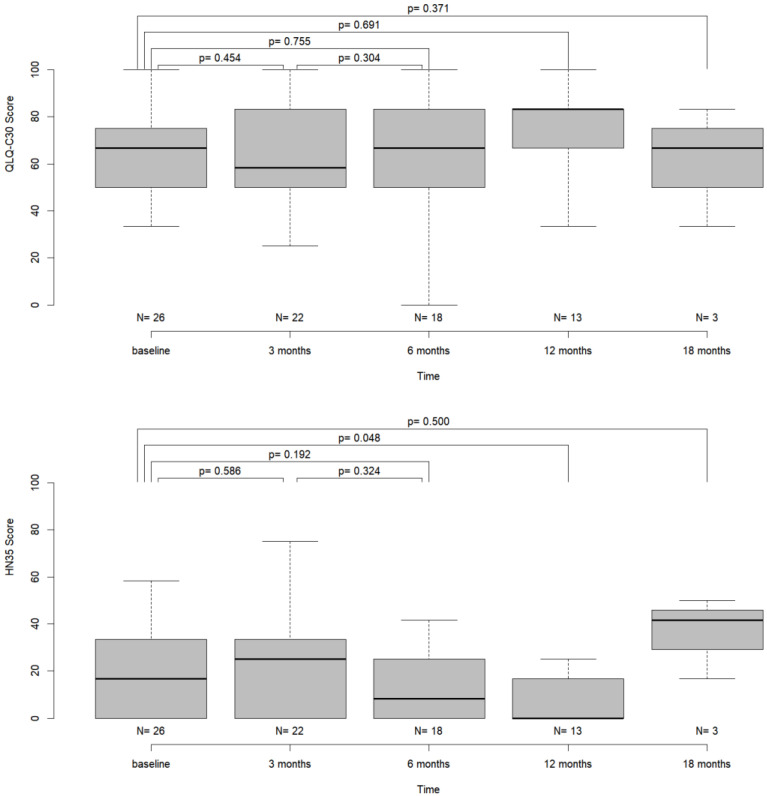
Boxplot diagram of the items “global health” and “pain” over time together with Wilcoxon test *p*-values for the pairwise comparison of time points.

The values for overall quality of life did not differ significantly from the baseline values at any time point after surgery (Wilcoxon test, *p*-values: 0.371–0.755) (Table 3). Figure 4 displays the dynamics of mean overall QoL values for the investigated cohort.

The scores for the “pain” item did not alter significantly after surgical therapy and during follow-up, apart from a decrease of the values 12 months after surgery (Wilcoxon-test; *p* = 0.048) indicating a reduction in pain (Table 3, Figure 4).

**Figure 5:**
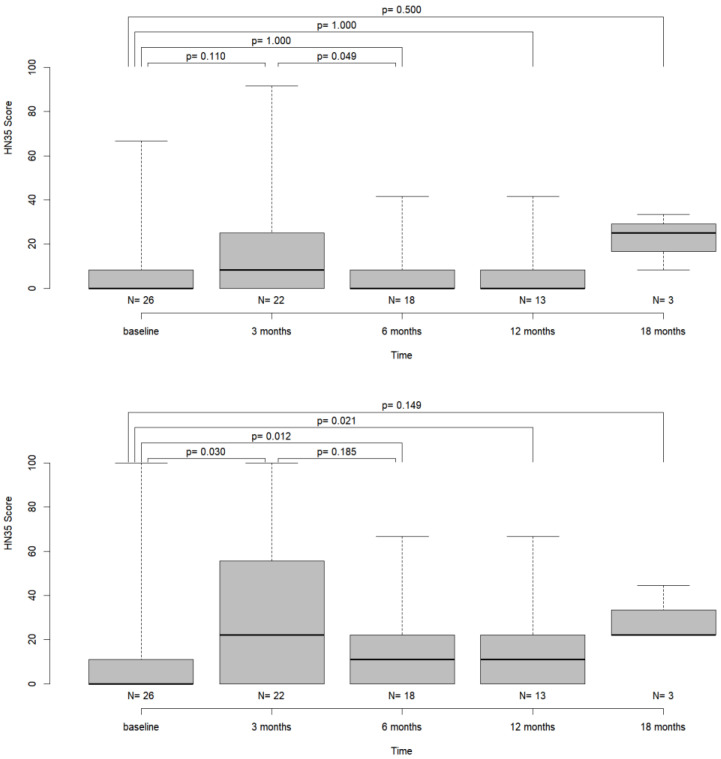
Boxplot diagram of the items “swallowing” and “speech” over time together with Wilcoxon test *p*-values for the pairwise comparison of time points.

Furthermore, the scores for “swallowing” showed a significant decrease between t1 and t2 (Wilcoxon-test; *p* = 0.049). All other comparisons of time points did not show significantly differing scores (Table 3, Figure 5).

The scores of the “speech” item showed a significant increase at 3 months (Wilcoxon test, *p* = 0.03) compared to baseline, indicating increased subjective impairment of speech. While the scores decreased at t2 and t3, values differed significantly between baseline and t3 (Wilcoxon test, *p* = 0.021, Table 3, Figure 4).

## 4. Discussion

While surgery has been broadly accepted as primary treatment for resectable SCCs of the oral cavity, reconstructive procedures are still a matter of debate, especially in early-stage tumors. Key arguments against free-flap reconstruction in early-stage tumors are longer surgery times, potential donor-site morbidity, and inferior function in terms of speech and swallowing.

Short surgery times are of high importance to minimize patient related peri- and postoperative complications, to shorten waiting times for patients and, thus, to reduce treatment delay and to maintain operation room (OR) efficiency. While reduction in treatment delay is of crucial prognostic relevance especially in patients suffering from early-stage tumors, our data clearly indicate that free-flap reconstruction does not necessarily lead to inefficient OR management with mean surgery times of close to 300 min for microvascular reconstructions in our cohort. Surgical procedures are inarguably faster when primary wound closure is chosen, but this sole factor should not be guiding decision making in this highly relevant question. Oncological safety and postoperative quality of life (QoL) are the essential outcome measures that should determine therapy planning and choice of procedures.

In the presented study, we assessed QoL in terms of global health, pain, and function by means of speech and swallowing. Several questionnaires for the assessment of quality of life are available, and, inarguably, there are strengths and frailties to each one, including the ones chosen for the presented investigation. While there may be instruments to assess QoL with a more detailed focus on single functional aspects following surgical procedures in the head and neck area, the successful usage of the QLQ-C30 and H&N35 questionnaires in several other studies confirms the validity of this method and enhances the comparability with other studies [16,20,21,22,23,24].

While the surgical and organizational advantages and disadvantages of free-flap reconstruction are often used as key arguments in the debate concerning reconstructive procedures in patients with early-stage tumors, postoperative quality of life should be the main factor guiding treatment decisions. Most available publications on the topic are based on retrospective analyses without preoperative QoL assessment. Broad comparability is further limited due to different tumor stages, questionnaires, and timing of QoL assessment. Hence, statements concerning postoperative QoL in dependence of different reconstructive procedures are contradictory, with several authors stating either superiority of primary wound closure or free-flap reconstruction. In most of the reported cases, the authors focused on tumors of the tongue, a fact that further reduces the universal validity of the reported data [10,11,13,25].

In most scores, we did not find significant changes between the pre- and postoperative values, except for the “pain”, the “swallowing” and the “speech” items. The significant reduction in pain after tumor resection has been shown by other studies and may serve as an argument in favor of surgical therapy in general [16,26]. Regarding the “speech” item, patients reported a significant subjective impairment of function postoperatively. The reported values improved during follow-up, but the scores still differed significantly from the baseline measurement after 12 months (Table 3 and Figure 4). While Bressmann et al. reported superior intelligibility in patients after free-flap reconstruction, other authors report better postoperative function after primary closure or secondary healing without wound closure [10,11,27]. Here, again, the focus often lies on tumors of the tongue, aggravating comparability with our results. Meanwhile, impairment of speech and swallowing function as seen in our analysis may be attributable to postoperative swelling and was reversible as demonstrated by not significantly differing score values between baseline and 18 months after surgical therapy. Longer follow-up and a larger cohort are needed in order to strengthen the validity of the data.

Furthermore, oncological outcome of the cohort was analyzed. Overall survival rates of patients with HNSCC in a “localized stadium” have been reported to range between 79 and 83% [28,29]. Here, the aim of treating specialists should be to further improve survival rates, and secondly, to provide a therapy with good postoperative quality of life in terms of functional and aesthetic outcomes. In our analysis, only two cases of local recurrence occurred during follow-up. While a median follow-up time of 12.5 months does not allow for a final evaluation, still the low rate of disease recurrence seems encouraging as many cases of locoregional recurrence develop within the first 12 to 18 months after primary treatment [16,30]. Safe surgical margins have been shown to be crucial for the occurrence of local disease recurrence and Lu et al. concluded that free-flap reconstruction in patients with SCC of the tongue resulted in wider margins and better progression-free survival compared to patients without free-flap reconstruction [9]. Before 2010, our in-house strategy implied the use of local reconstruction and split skin grafts for defect closure after tumor resection. A retrospective analysis presented a not negligible rate of local recurrences [31]. The analysis showed 122 OSCC after clinical staging (certainty level 2), of which 116 patients received local reconstruction. After pathological staging (certainty level 4), 5-year disease-free survival was 61.6% in stage I and II OSCCs [31]. Considering the oncological outcome in the literature and our own experience with local reconstruction, we feel reinforced with our consistent microvascular therapeutic strategy.

There are frailties to the study design that must be discussed. The statistical power of an unrandomized one-armed observational study is limited in the first place, as the primary aim of the study was to evaluate the hypothesis of good functional and oncological outcome after tumor resection and free-flap reconstruction in patients with early-stage OSCC. Secondly, the overall number of patients and the follow-up time are yet limited, and therefore the presented results may be regarded as a promising tendency at the most. This applies to the oncological results as well as to the reported functional outcomes, as only three patients completed the questionnaires at time point 4 (18 months after surgery). Here, another evaluation of the results is needed after all included patients completed the whole follow-up period of 24 months. Furthermore, the patient cohort is heterogeneous regarding tumor localization within the oral cavity. The functional advantages or disadvantages of free-flap reconstruction however are possibly mostly dependent on the anatomical subunit affected by the tumor and thus by the method of reconstruction, as stated above. Here, again, more patients are warranted to enable a subsite-specific analysis of postoperative function and QoL. We therefore plan to extend the study on additional cancer centers to enhance patient numbers and to add value to the data after the protocol has been proven to be feasible. In a further step, we plan to implement randomization in two treatment groups (primary wound closure vs. free-flap reconstruction) in a follow-up study (Supplementary Materials, Figure S1).

## 5. Conclusions

The presented study successfully demonstrates that free-flap reconstruction in early-stage OSCC is a safe and efficient procedure that provides convincing oncological results while preserving a high quality of life. Surgery time and duration of hospitalization are moderate and comparable to patients receiving primary wound closure. To further enhance the quality of the data, the presented study will be extended to a multicentric approach.

## Figures and Tables

**Table 1:** Epidemiological and pathological characteristics of the investigated cohort (T Stadium: Tumor Stadium 1–4; N Stadium: Cervical lymph node Stadium 0–3; M Stadium: Distant metastases Stadium 0–1; UICC: Union internationale contre le cancer—Stadium 1–4, R: Resection Status 0–1).

Parameter	n (%)
Gender	
Female	10 (38.5)
Male	16 (61.5)
Age	
<65 years	13 (50)
>65 years	13 (50)
Pathological T Stadium	
T1	18 (69.2)
T2	8 (30.8)
T3	-
T4	-
N Stadium	
0	26 (100)
1	-
2a	-
2b	-
2c	-
3	-
M Stadium	
0	26 (100)
1	0
UICC	
1	18 (69.2)
2	8 (30.8)
3	-
4	-
Differentiation Grade	
1	6 (23.1)
2	14 (53.8)
3	2 (7.7)
Missing	4 (15.4)
R	
0	26 (100)
1	-
Localization	
Floor of the mouth	5 (19.2)
Tongue	11 (42.3)
Maxilla	5 (19.2)
Buccal Plane	5 (19.2)
Disease Recurrence during follow-up	
Yes	2 (7.7)
No	24 (92.3)

**Table 2:** Perioperative morbidity and hospitalization.

Parameter	n (%)
Tracheostomy	10 (38.5%)
Duration of Tracheostomy	11.2 ± 7.2 days
Gastrostomy feeding tube	2 (7.7%)
Neck Dissection	
Unilateral	12 (46.2%)
Bilateral	14 (53.8%)
Hospitalization	Mean duration (days)
ICU/IMC	4.65 ± 5.5
Overall	13.1 ± 7.6

**Table 3:** Median scores and quantile range of the QLQ-C30 and H&N 35 scores at different time points during the study period.

Item	t0 (*n* = 26)	t1 (*n* = 22)	t2 (*n* = 18)	t3 (*n* = 13)	t4 (*n* = 3)
Global Health	66.7 (50–75)	58.3 (50–83.3)	66.7 (52.1–83.3)	83.3 (66.7–83.3)	66.7 (50–75)
Pain	16.7 (2.1–31.2)	25 (0–33.3)	8.3 (0–22.9)	0 (0–16.7)	41.7 (29.2–45.8)
Swallowing	0 (0–8.3)	8.3 (0–22.9)	0 (0–8.3)	0 (0–8.3)	25 (16.7–29.2)
Speech	0 (0–11.1)	22.2 (0–52.8)	11.1 (0–22.2)	11.1 (0–22.2)	22.2 (22.2–33.3)

## Data Availability

The data presented in this study are available on request from the corresponding author.

## Supplementary Materials

The following supporting information can be downloaded at: https://www.mdpi.com/article/10.3390/jcm12144833/s1, Figure S1. Flow chart depicting the future study protocol including patient randomization.
