# Supplementary figures and images for: Free-Flap Reconstruction in Early-Stage Squamous Cell Carcinoma of the Oral Cavity—A Prospective Monocentric Trial to Evaluate Oncological Outcome and Quality of Life
Source: J Clin Med. 2023 Jul 22;12(14):4833. doi: 10.3390/jcm12144833 (PMC10381471; doi:10.3390/jcm12144833)

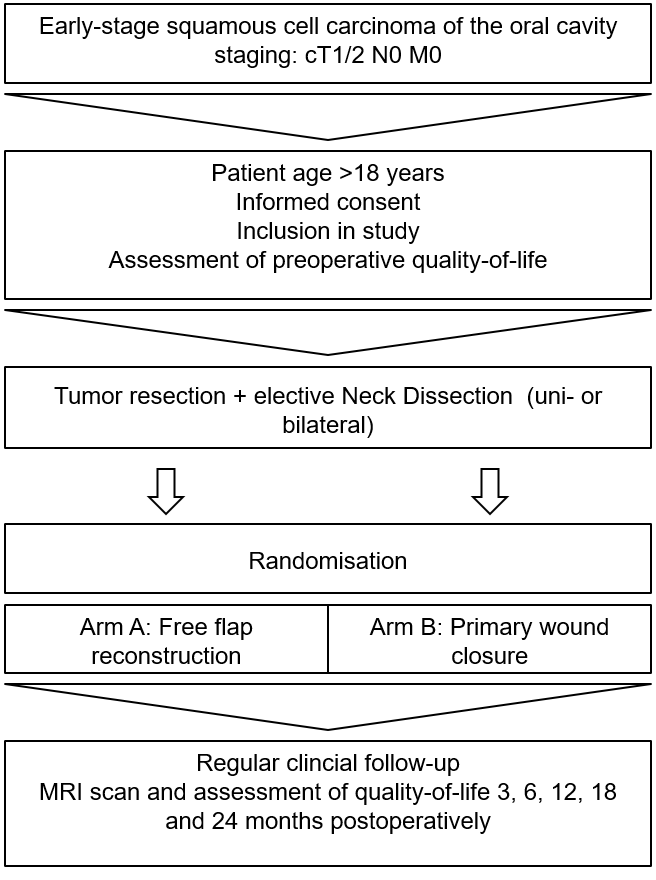

Supplement: Supplementary file 1 [file jcm-12-04833-s001.zip › jcm-2439024-supplementary.tif]
